# Long‐read sequence capture of the haemoglobin gene clusters across codfish species

**DOI:** 10.1111/1755-0998.12955

**Published:** 2018-12-04

**Authors:** Siv Nam Khang Hoff, Helle T. Baalsrud, Ave Tooming‐Klunderud, Morten Skage, Todd Richmond, Gregor Obernosterer, Reza Shirzadi, Ole Kristian Tørresen, Kjetill S. Jakobsen, Sissel Jentoft

**Affiliations:** ^1^ Centre for Ecological and Evolutionary Synthesis Department of Biosciences University of Oslo Oslo Norway; ^2^ Roche NimbleGen Inc. Madison Wisconsin; ^3^ Roche Diagnostics Mannheim Germany; ^4^ Roche Diagnostics Oslo Norway

**Keywords:** codfishes, comparative genomics, Gadiformes, PacBio sequencing, targeted sequence capture, teleosts

## Abstract

Combining high‐throughput sequencing with targeted sequence capture has become an attractive tool to study specific genomic regions of interest. Most studies have so far focused on the exome using short‐read technology. These approaches are not designed to capture intergenic regions needed to reconstruct genomic organization, including regulatory regions and gene synteny. Here, we demonstrate the power of combining targeted sequence capture with long‐read sequencing technology for comparative genomic analyses of the haemoglobin (*Hb*) gene clusters across eight species separated by up to 70 million years. Guided by the reference genome assembly of the Atlantic cod (*Gadus morhua*) together with genome information from draft assemblies of selected codfishes, we designed probes covering the two *Hb* gene clusters. Use of custom‐made barcodes combined with PacBio RSII sequencing led to highly continuous assemblies of the LA (~100 kb) and MN (~200 kb) clusters, which include syntenic regions of coding and intergenic sequences. Our results revealed an overall conserved genomic organization of the *Hb* genes within this lineage, yet with several, lineage‐specific gene duplications. Moreover, for some of the species examined, we identified amino acid substitutions at two sites in the *Hbb1* gene as well as length polymorphisms in its regulatory region, which has previously been linked to temperature adaptation in Atlantic cod populations. This study highlights the use of targeted long‐read capture as a versatile approach for comparative genomic studies by generation of a cross‐species genomic resource elucidating the evolutionary history of the *Hb* gene family across the highly divergent group of codfishes.

## 1 INTRODUCTION

The rapid advancement of high‐throughput sequencing has over the last decade revolutionized genomic research with the increasing numbers of whole‐genome resources available for multiple vertebrate species, including the diverse group of teleost fishes (Ellegren, 2014; Goodwin, McPherson, & McCombie, 2016; Malmstrøm, Matschiner, Tørresen, Jakobsen, & Jentoft, 2017; Malmstrøm et al., 2016; Volff, 2004). For investigations concerning specific genomic regions, however, there is no need for complete genome information, which has spurred the development of reduction complexity approaches such as targeted sequence capture (Grover, Salmon, & Wendel, 2012; Samorodnitsky et al., 2015; Teer et al., 2010). The basic idea of targeted sequence capture involves design of specific probes covering the particular genomic area of interest generating an enriched coverage of the targeted sequences in a cost‐ and time‐efficient manner (Grover et al., 2012; Turner, Ng, Nickerson, & Shendure, 2009). Thus, target capture is an attractive method for larger investigations of specific genomic regions from multiple individuals, that is, population studies and/or across closely related species (Jones & Good, 2015; Wang et al., 2015). Most studies using targeted sequence capture have to a large extent been directed towards the exome, often supported by the existence of a reference genome (Broeckx et al., 2014; Yoshihara et al., 2016), or transcriptome assemblies (Syring et al., 2016). Recent reports have, however, been focusing on off‐target sequences in noncoding regions (Guo et al., 2012; Morin et al., 2016; Syring et al., 2016; Yoshihara et al., 2016), as they may contain crucial regulatory elements varying in sequence and length between populations or species and could be of functional and evolutionary importance (Woolfe et al., 2004).

To date, comparative studies using sequence capture have been mainly based on short‐read sequencing technology and probe design targeting genic regions (Bragg, Potter, Bi, & Moritz, 2015; George et al., 2011; Li et al., 2015; Samorodnitsky et al., 2015). Consequently, construction of continuous sequences enabling resolution of gene organization across species has not yet been looked into. Comparative genomic studies of gene organization or synteny requires longer, more continuous stretches of DNA containing more than one gene (Huddleston et al., 2014). By its ability to span long stretches of repeats, long‐read sequencing technology has been successfully applied to improve genome assembly statistics and generation of highly continuous genome assemblies for a growing number of species (English et al., 2012; Kim et al., 2014; Lin et al., 2016; Tørresen et al., 2017, 2018). Incorporation of long PacBio reads has for instance resulted in a significantly improved version of the Atlantic cod (*Gadus morhua*) genome assembly, that is, a 50‐fold increase in sequence continuity and a 15‐fold reduction in the proportion of gap (Tørresen et al., 2017). Furthermore, recent studies report the combination of capture and long‐read sequencing as highly efficient in enriching and assembly of full‐length complex genes as well as detailed characterization of chromosomal structural variations (Giolai et al., 2017; Wang et al., 2015; Witek et al., 2016). Correspondingly, utilizing long‐read sequencing technology in combination with targeted capture could yield longer continuous assemblies of specific genomic regions of interest, allowing in‐depth comparative genomic studies including synteny analyses, in species where reference genomes are not available.

In fishes, the haemoglobin (*Hb*) gene family, encoding the protein subunits Hba and Hbb, has shown to be of importance for ecological adaptation, as environmental factors such as temperature directly influence the ability of Hb to bind O_2_ at respiratory surfaces and its subsequent release to tissues (Wells, 2005). In a recent report, a characterization of the *Hb* gene repertoire by comparative draft genome analysis uncovered a remarkably high *Hb* gene copy variation among species of codfishes (Baalsrud et al., 2017). Based on the gene copy number, a negative correlation between the number of *Hb* genes and depth of which the species occur was observed, as well as signs of diversifying selection on the gene paralogues suggesting that the variable environment in epipelagic waters, has facilitated a larger more diverse *Hb* gene repertoire (Baalsrud et al., 2017). However, the rather fragmented draft genomes did not allow for reconstruction of the gene organization and a deeper understanding of the evolution of the *Hb* clusters.

Moreover, two tightly linked polymorphisms at amino acid positions 55 and 62 of the Hbb1‐globin suggested associated with thermal adaptation have been demonstrated in Atlantic cod populations. These polymorphisms exhibit a latitudinal cline in allele frequency in populations inhabiting varying temperature and oxygen regimes for Atlantic cod in the North Atlantic and Baltic Sea (Andersen et al., 2009). Populations found in the southern regions display the Hbb1‐1 variant (Met55Lys62), whereas more northern populations largely display the Hbb1‐2 variant (Val55Ala62) (Andersen et al., 2009). The Hbb1‐1 variant has been proposed by protein modelling to be insensitive to temperature, whereas Hbb1‐2 is temperature‐dependent with a higher O_2_ affinity than Hbb1‐1 at colder temperatures (Andersen et al., 2009). However, this assumption has recently been questioned by Barlow, Metcalfe, Righton, & Berenbrink, 2017, where in vitro experimental testing shows inconsequential results with respect to haemoglobin–oxygen binding and temperature sensitivity of haemoglobin–oxygen binding of these two Hbb1 variants.

Additionally, an indel polymorphism within the promoter of the *Hbb1* gene has been reported to be in linkage disequilibrium with the above‐mentioned polymorphisms (Star et al., 2011). Examination of multiple Atlantic cod populations uncovered that a longer promoter variant is associated with *Hbb1‐2* and found to upregulate its gene expression at higher temperatures, that is, aiding in the maintenance of total oxygen‐carrying capacity (Star et al., 2011).

In teleosts, the *Hb* genes are found to reside at two distinct genomic regions, named the MN and LA clusters according to the genes that flank the *Hbs* (e.g., Hardison, 2012; Opazo, Butts, Nery, Storz, & Hoffmann, 2012). Earlier reports have shown that there is a high evolutionary turnover of *Hb* genes across teleosts, with lineage‐specific duplications and losses, which is in stark contrast to genes flanking the *Hb* genes, where the synteny is highly conserved (Feng et al., 2014; Opazo et al., 2012; Quinn et al., 2010). In this study, the overall goal was to elucidate the evolutionary past of the *Hb* clusters—including *Hb* genes, flanking genes and intergenic sequences—within the phylogenetically diverse group of codfishes (Gadiformes) by taking advantage of long‐read sequencing technology combined with targeted sequence capture. Eight codfish species were carefully selected on the basis of both phylogenetic and habitat divergence, implying that they are exposed to a variety of environmental factors as well as displaying distinct life‐history traits. A highly continuous genome assembly of Atlantic cod (Tørresen et al., 2017) as well as low‐coverage draft genome assemblies of all eight species (Malmstrøm et al., 2016, 2017) was used in the design of the probes covering the genomic regions of interest. To enable targeted sequence capture for PacBio RSII sequencing, we modified the standard protocol for sequence capture offered by NimbleGen, the SeqCap EZ (Roche NimbleGen), an in‐solution capture hybridization protocol, as well as generated custom‐made barcodes. This combined approach resulted in successful capturing and assembling of the two *Hb* gene clusters across the codfishes examined. The generation of highly continuous assemblies—for most of the species—enabled reconstruction of micro‐synteny revealing lineage‐specific gene duplications and identification of a relatively large and interspecies variable indel located in the promoter region between the *Hbb1* and *Hba1* genes.

Our study demonstrates that combining sequence capture technology with long‐read sequencing is a highly efficient and versatile method to investigate specific genomic regions of interest—with respect to micro‐synteny, regulatory regions and genetic organization—across distantly related species where genome sequences are lacking.

## 2 RESULTS

### 2.1 Capture and de novo assembly of the LA and MN regions

**Figure 1:**
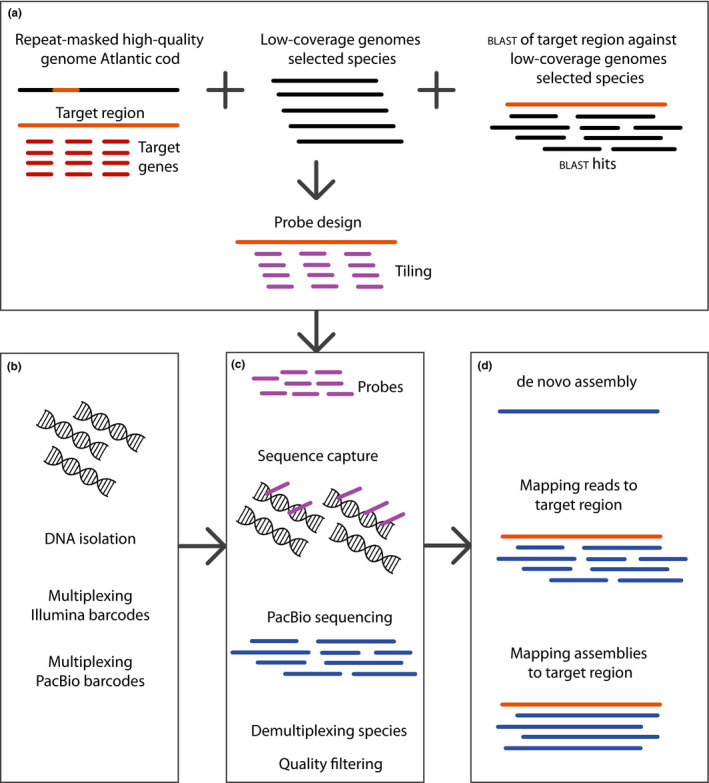
Flowchart of sequence capture approach. (a) Sequence data from the Atlantic cod genome (Tørresen et al., 2017; gadMor2) combined with gene sequences of target genes and sequences from low coverage genomes of the additional codfishes are combined to generate probes. (b) Isolated DNA is multiplexed with Illumina and PacBio barcodes. (c) Raw reads for each species are used to score all probes, ensuring that no repeated sequences are present. DNA probes are used in solution on isolated DNA for all of the included species, hybridizing to the target sequences. Target sequences are then captured and sequences on the PacBio RSII sequencing platform. (d) Downstream bioinformatics includes demultiplexing of reads and trimming, making the reads ready for downstream analysis such as mapping and de novo assembly

**Figure 2:**
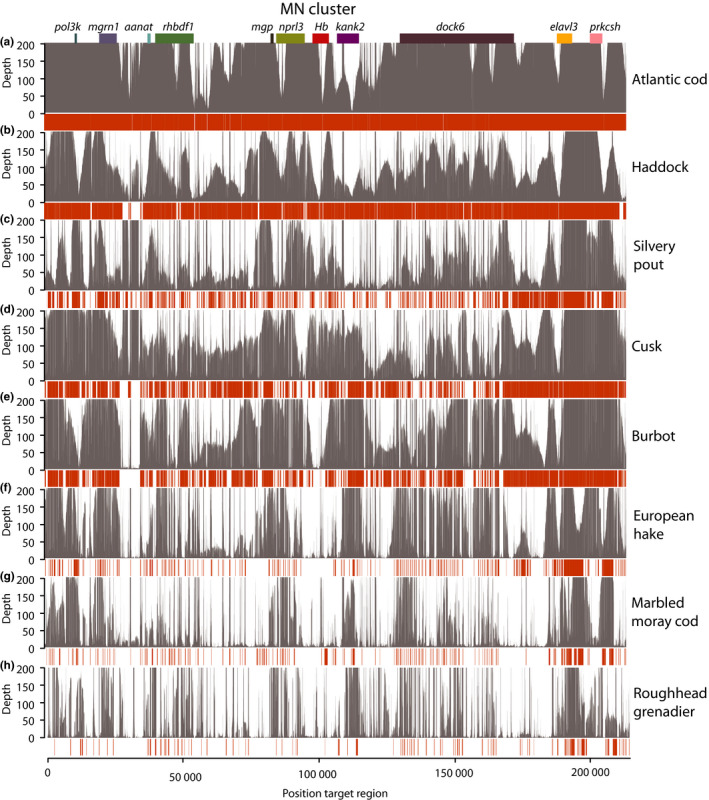
Mapping of reads and assemblies against the MN target region. Each panel shows the reads and de novo assembly mapped against the MN target region in grey and orange, respectively, for the selected species included in this study, that is, Atlantic cod, haddock, silvery pout, cusk, burbot, European hake, marbled moray cod and roughhead grenadier. The positions of genes in the target region are indicated at the top

**Figure 3:**
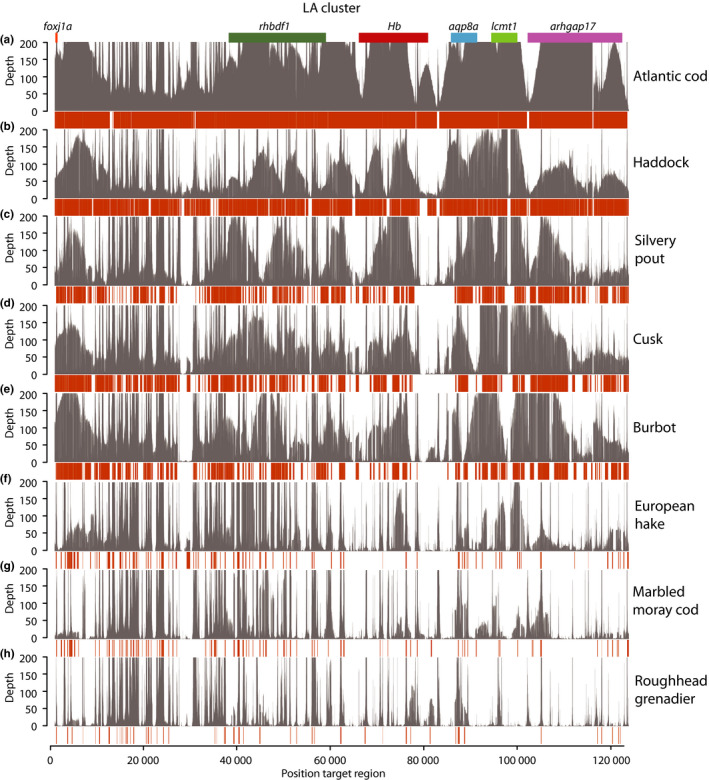
Mapping of reads and assemblies against the LA target region. Each panel shows the reads and de novo assembly mapped against the LA target region in grey and orange, respectively, for the selected species included in this study, that is, Atlantic cod, haddock, silvery pout, cusk, burbot, European hake, marbled moray cod and roughhead grenadier. The positions of genes in the target region are indicated at the top

**Figure 4:**
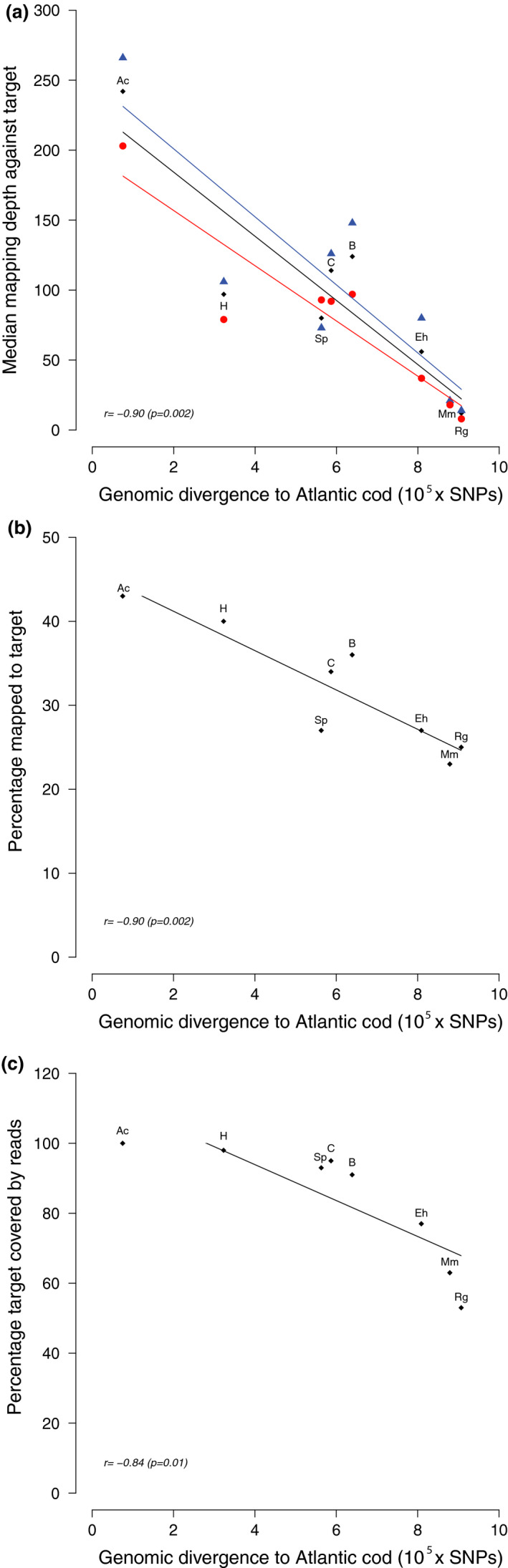
The relationship between capture success and genomic divergence to Atlantic cod. Linear regression of the relationship between the genomic divergence to Atlantic cod (SNPs × 10^5^) and (a) median mapping depth for the MN region (blue), LA region (red) and the combined target regions (black); (b) the percentage of reads mapping to the target regions; (c) the percentage of the target regions covered by reads to a minimum depth of 10×. For each regression, the correlation coefficient, *r*, is shown along with a *p*‐value. Each data point is labelled by species according to this code: Ac = Atlantic cod, H = haddock, Sp = silvery pout, C = cusk, B = burbot, Eh = European hake, Mm = marbled moray cod and Rg = roughhead grenadier

The probe design (workflow schematically shown in Figure 1) resulted in a total of 7,057 capture probes based on the target regions in Atlantic cod, covering 337 kbp of sequence. 26,774 probes were designed for the additional codfishes, covering a total of 1.82 Mbp of target sequence. The *Hb* gene clusters (LA and MN) were successfully captured and enriched for eight codfishes (see Table S8); Atlantic cod (*Gadus morhua*), haddock (*Melanogrammus aeglefinus)*, silvery pout (*Gadiculus argenteus*), cusk (*Brosme brosme*), burbot (*Lota lota*), European hake (*Merluccius merluccius*), marbled moray cod (*Muraenolepus marmoratus*) and roughhead grenadier (*Macrourus berglax*), with number of reads spanning from 35,573 to 73,005 (Table 1). The average read length was 3,032 bp, varying from 2,836 bp in European hake to 3,265 bp in burbot, resulting in the capture of an average of 16.71 Mbp per species (Table 1). By mapping reads back to the Atlantic cod target regions, we found that the average mapping depth (i.e., coverage) was variable across the LA and MN regions for all species (Figures 2 and 3). Because of the skewed distribution of mapping depth, we also calculated median depth, which was, as expected, the highest for Atlantic cod at 242x (Table S1). The median mapping depth was consistently high for most of the other species as well, with the lowest for roughhead grenadier (12x). Both median and average depths for the MN region were persistently higher than for the LA region for all species, with the exception of silvery pout (Table S1). Furthermore, positions with high degree of mapping corresponded to the location of the genes used in the design of the capture probes across all species (Figures 2 and 3). The percentage of reads mapping to the target regions ranged from 25 to 43%; however, the percentage of the target regions covered by reads ranged from 53 to 100% with five species having more than 90% of the target regions covered by reads (Figure 4c and Table S1).

**Table 1:** Number of reads and bases captured and sequenced for each species, number of unitigs (utg), largest utg and N50 values for the assemblies

Species	Latin name	Number of reads	Number of bases	Number of utgs	Largest utg (bp)	N50 (bp)
Atlantic cod	*Gadus morhua*	73005	217252583	278	79,020	7,728
Haddock	*Melanogrammus aeglefinus*	35573	107839552	227	52,433	7,227
Silvery pout	*Gadiculus argenteus*	69775	212519845	410	35,801	7,098
Cusk	*Brosme brosme*	55348	175883008	394	64,145	7,322
Burbot	*Lota lota*	56155	165360828	205	70,602	8,055
European hake	*Merluccius merluccius*	65661	180558336	311	31,558	6,523
Marbled moray cod	*Muraenolepus marmoratus*	52076	148100933	455	30,019	6,632
Roughhead grenadier	*Macrourus berglax*	46195	129085001	325	35,216	7,122

To address factors influencing capture success, we compared various capture statistics to overall genomic divergence between the Atlantic cod genome and independent WGS data for each species from (Malmstrøm et al., 2016, 2017) (Table S1). We found a strong negative correlation between genomic divergence to Atlantic cod and median mapping depth against the target regions (*r* = −0.90, Figure 4a), percentage of reads mapped to the target regions (*r* = −0.90, Figure 4b) and percentage of reads mapped to the target regions (*r* = −0.84, Figure 4c).

We constructed de novo assemblies with quite consistent assembly statistics across species. Contig N50 ranged from 8,055 bp in burbot to 6,523 bp in European hake and the total number of contigs varied from 205 in burbot to 455 in marbled moray cod. However, there was some variation in the size of the largest contig, which ranged from 79 kbp in Atlantic cod to 30 kbp in marbled moray cod (Table 1). To evaluate whether the assemblies represent the actual target regions, we mapped the de novo assemblies for each species to the target regions in Atlantic cod, for which the capture design is largely based upon (Figures 2 and 3). As expected, the assemblies corresponded to the regions with high coverage of reads, that is, the areas of the target regions containing genes included in the probe design.

### 2.2 Organization of the *Hb* gene regions

**Figure 5:**
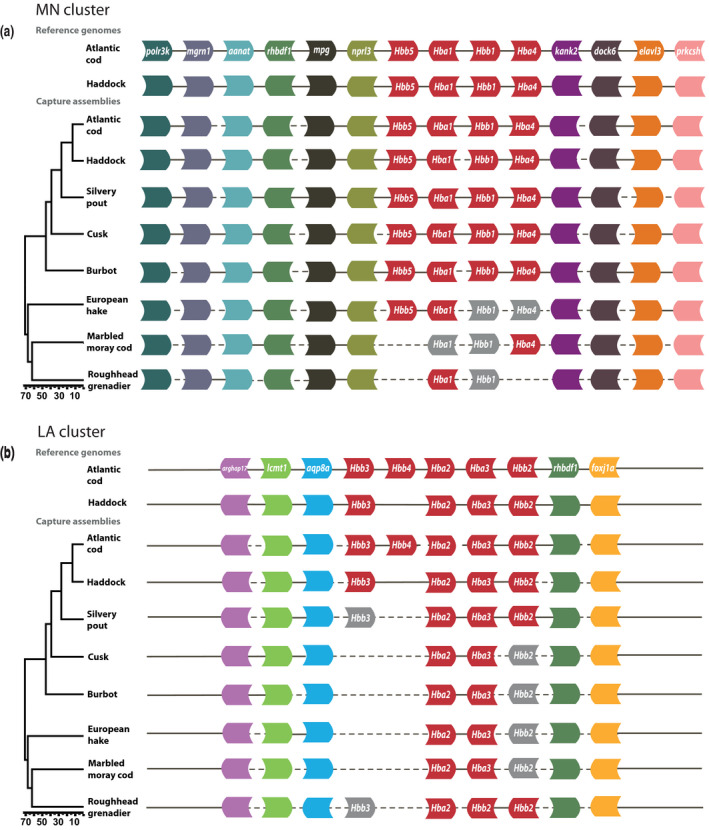
Organization of the *Hb* gene clusters. At the top, the reconstructed micro‐synteny of the haemoglobin gene clusters is shown based on the genomes of Atlantic cod (Tørresen et al., 2017; gadMor2) and haddock (Tørresen et al., 2018; melAeg). Below, we illustrate the genomic synteny inferred from the de novo assemblies for the eight species included in the capture experiment, mapped on a time‐calibrated species phylogeny modified from Malmstrøm et al. (2016) with time given in million years (Ma). Stippled lines indicate assembly gaps; here, we assume that the orientation of genes corresponds to the genomes of Atlantic cod and haddock. Gray boxes indicate genes that have been identified in Baalsrud et al. (2017) but are absent in the de novo assemblies. (a) Display the micro‐synteny across the MN region. (b) Display the micro‐synteny across the LA region

Our capture design combined with long‐read PacBio sequencing allowed us to reconstruct micro‐synteny of the MN and LA regions for Atlantic cod, haddock, silvery pout, cusk, burbot, European hake, marbled moray cod and roughhead grenadier (Figure 5). From the de novo assemblies, we were able to identify the majority of the *Hb* genes and all of the flanking genes, which show that our capture design was successful. However, the degree of continuity varied in the different assemblies. In Atlantic cod, haddock, silvery pout, cusk, burbot and European hake, we could infer micro‐synteny revealing that *Hb* and their flanking genes organization largely followed what has previously been reported for Atlantic cod (Figure 5) (Star et al., 2011). We found *Hbb4* only to be present in Atlantic cod (Figure 5b)*,* which is in line with Baalsrud et al., 2017. Furthermore, the de novo assemblies confirmed a linage‐specific duplication of *Hbb2* in the roughhead grenadier (Baalsrud et al., 2017). Additionally, we identified a complete *Hba4*‐like gene in the assembly of the marbled moray cod, not earlier identified in this species. However, the *Hba4‐*like gene in marbled moray cod is likely a pseudogene due to a frameshift mutation causing multiple stop codons. Furthermore, we were able to identify most of the *Hb* genes reported in the recent study by Baalsrud et al. (2017); however, a few are missing from our data set (Figure 5a,b). Pairwise sequence alignment of these paralogous *Hb* genes from Baalsrud et al. (2017) revealed sequence identities up to 98% (Table S2).

### 2.3 Target regions in the haddock and Atlantic cod genome assemblies

As a proof of concept, we reconstructed the micro‐synteny of the target regions in the recent genome assemblies of Atlantic cod (Tørresen et al., 2017; gadMor2) and haddock (Tørresen et al., 2018; melAeg). In Atlantic cod, the MN region is located on linkage group 2 (Figure 5a) and LA on linkage group 18 (Figure 5b); in haddock, MN is located on scaffold MeA_20160214_scaffold_771 (Figure 5a) and LA on scaffold MeA_20160214_scaffold_1676 (Figure 5b). The overall gene organization in Atlantic cod was congruent with Wetten et al. (2010) except for the relative direction of the genes *foxj1a* and *rhbdf1*. Furthermore, the organization of *Hb*s and their flanking genes in the genome assembly of haddock is conserved compared to Atlantic cod with the exception of *Hbb4* in the MN region, which is absent in haddock (Figure 5).

### 2.4 Repetitive sequences in the *Hb* gene regions

Quantifying the amount of repetitive sequences in the target regions was only possible for Atlantic cod (gadMor2) and haddock (melAeg), for which high‐quality genome assemblies exist. The amount of repetitive sequences in the target regions differed between the MN cluster and the LA cluster in Atlantic cod. The MN region (214 kb) contained a total of 10.7% repeated sequences, including 1.0% retro‐elements, 1.3% transposons, 5.8% simple repeats and 2.6% of various low‐complexity and unclassified repeated sequences (Table S3). In comparison, in the smaller LA region (123 kb), the proportion of repeated sequences was twice as high (20.3%), which comprised of 2.8% retro‐elements, 2.4% transposons, 13.8% simple repeats and 1.3% of various low‐complexity and unclassified repeated sequences. Furthermore, the orthologous target regions in haddock followed the same pattern. The MN region contained 16.3% repeated sequences, in contrast to 19.8% found in the LA region (Table S3).

### 2.5 Insertions and deletions in the promoter region of *Hba1*–*Hbb1*

**Figure 6:**
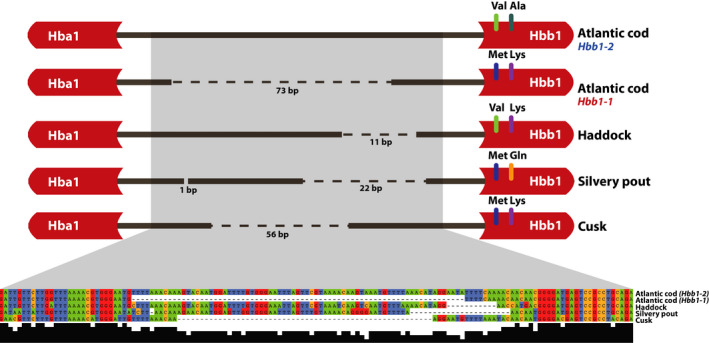
Polymorphisms in the bidirectional promoter between *Hba1* and *Hbb1* for five species in the Gadidae family. A schematic representation of *Hba1* and *Hbb1* with the promoter region between them. The region contains an indel polymorphism of variable length across the five species, as indicated by gaps. For each species/variant, the alignment is shown along with amino acid substitutions at positions 55 and 62 in the translated part of the *Hbb1* gene

The previously shown 73‐bp indel in the bidirectional promoter region of *Hba1* and *Hbb1*—discerning the cold‐adapted migratory Northeast Artic (NEA) cod from the more temperate‐adapted southern Norwegian coastal (NC) cod (Star et al., 2011)—was confirmed by the improved version of the NEA cod assembly (gadMor2). The continuity of our capture assemblies (Figure 5) enabled location of the orthologous captured regions in haddock, silvery pout and cusk. In each of the species, an indel of variable length was identified (Figure 6). Compared to the long promoter variant—found to be linked with the *Hbb1‐2* in Atlantic cod—the indel is shorter in the other species by 11 bp in haddock, 22 bp in silvery pout and 56 bp in cusk (Figure 6). Although the indels are varying in length, the conserved flanking sequences in the alignment clearly show that they represent orthologous regions. Moreover, we found the amino acid positions at 55 and 62 of the *Hbb1* gene to vary between species; Haddock has Val55‐Lys62, silvery pout has Met55‐Gln62, while cusk has Met55‐Lys62 similar to NEA cod (Figure 6). Additionally, we investigated amino acid positions 55 and 62 in the *Hbb1* gene across a number additional codfish species for which we have available gene sequences from Baalsrud et al. (2017), revealing these sites to be variable across this lineage (Table S4). Ancestral reconstruction of *Hbb1* demonstrated that the ancestral state in position 55 was Met in codfishes and in position 62 was Lys in all codfishes except *Bregmaceros cantori* (Supporting Information Figures S1 and S2).

## 3 DISCUSSION

### 3.1 Capture of *Hb* gene clusters with 70 million years divergence time reveal conserved gene synteny and lineage‐specific *Hb* duplications

We here demonstrate a successful in‐solution targeted sequence capture and assembling of coding and noncoding sequences of the *Hb* clusters from codfish species separated by up to 70 million years (My) of evolution. Two features make our approach unique from earlier studies. First, the target regions consisted of both coding and noncoding genomic sequences. Second, we designed custom‐made probes in order to utilize the long‐read PacBio sequencing platform. In contrast to previous targeted capture sequencing studies based on short‐read sequencing technologies (George et al., 2011; Mascher et al., 2013), our approach enabled the generation of highly continuous assemblies of the *Hb* clusters across distantly related codfishes.

The organization and orientation of the *Hb* flanking genes that we identified were conserved across all species (Figure 5a,b). However, in concordance with earlier studies of the *Hb* regions, we found significant variation in copy numbers of the *Hb* genes, with linage‐specific duplications and losses (Baalsrud et al., 2017; Feng et al., 2014; Opazo et al., 2012; Star et al., 2011). We only found *Hbb4* in Atlantic cod, supporting earlier studies showing that *Hbb4* is the result of a recent duplication in this species (Baalsrud et al., 2017; Borza, Stone, Gamperl, & Bowman, 2009). Interestingly, the presence of two copies of *Hbb2* on the same contig in the roughhead grenadier de novo assembly confirmed a lineage‐specific gene duplication of *Hbb2* in this species. This duplication was postulated in recent study of *Hbs* in codfishes (Baalsrud et al., 2017), but due to the lack of gene synteny in the draft genome assemblies, it was not possible to determine with certainty. Additionally, a copy of the *Hba4* gene was found in the de novo assembly of the marbled moray cod not found in the previous study by Baalsrud et al. (2017). The presence of a frame‐shifting mutation that is causing multiple stop codons indicated that this *Hba4* gene is most likely a pseudogene. *Hba4* is also a pseudogene in the closely related species *Mora moro*,* Trachyrincus scabrus*,* T. murrayi* and *Melanonus zugmayeri* (Baalsrud et al., 2017). Although we identified most of the *Hb* genes from Baalsrud et al. (2017), a few were absent from this data set (Figure 5a,b), which we suspect may be due to collapse of paralogous *Hb* genes, as they may have as high as 98% sequence identity (Table S2).

### 3.2 Length variation in the bidirectional *Hba1‐Hbb1* promoter within the codfishes

The discovery of a promoter of variable length between *Hba1* and *Hbb1* in different species (Figure 6) was concordant with earlier findings of length variation in the homologous region in different populations of Atlantic cod (Star et al., 2011). The migratory NEA cod population has been shown to harbour the 73‐bp‐longer variant at a higher frequency compared to coastal cod populations (see Figure 6 and Star et al., 2011). Interestingly, we found relatively long promoters with high sequence similarity to the NEA cod indel in haddock and silvery pout. In contrast, cusk displayed a relatively short promoter, however, still 17 bp longer than in NC cod (Figure 6). Furthermore, we found the amino acid positions 55 and 62 in *Hbb1,* known to be polymorphic in Atlantic cod, to be variable across all codfishes included in this study (Figure 6). Investigations of the same positions in a number of additional codfishes for which we have available gene sequences (Baalsrud et al., 2017) revealed that these positions are highly variable across this linage (Table S4). Notably, the most likely ancestral state of codfish *Hbb1* is Met55Lys62 (Supporting Information Figures S1 and S2). Cusk and the coastal/southern Atlantic cod thus both display the ancestral state as well as a short promoter, although the cusk promoter was 17 bp longer (Figure 6). Collectively, these results suggest two different scenarios for promoter length evolution. Scenario 1: The short promoter represents the ancestral state of the Gadidae family (including cusk and Atlantic cod; see Malmstrøm et al., 2016) and that silvery pout and some populations of Atlantic cod have evolved a longer promoter. Scenario 2: The long promoter is the ancestral state with independent deletions of variable lengths in cusk, silvery pout, haddock and coastal/southern Atlantic cod (*Hbb1‐1*). To disentangle this, we would need to obtain promoter sequences from a broader spectrum of gadiform species as well as data at the population level for some of the same species. Moreover, in both scenarios, cusk and coastal/southern Atlantic cod (*Hbb1‐1*) have maintained the ancestral Met55Lys62, while silvery pout, haddock and NEA cod (*Hbb1‐2*) have acquired substitutions at these positions due to similar selection pressures or genetic drift. In this regard, it could be mentioned that the NEA cod, haddock and silvery pout display migratory behaviour (e.g., diurnally feeding movements as well as seasonal spawning migrations) compared to the more stationary cusk and coastal cod (Eschemeyer & Fricke, 2017) which could mean that they have a higher O_2_ demand and are exposed to greater temperature variation, which in turn has selected for a temperature‐dependent long promoter. Furthermore, given that promoter length and positions 55/62 at *Hbb1* are important genetic components of temperature adaptation in Atlantic cod populations (Star et al., 2011), they most likely play a role in temperature adaptation in the other codfishes.

### 3.3 Assembly success affected by probe design and repeat content

In some species, nearly the complete LA and MN regions were assembled in large contigs containing multiple genes including cusk, whereas in other species such as the more distantly related roughhead grenadier, the cluster is more fragmented (Figure 5). In all species, the areas of the target regions that harbour genes of which probes are designed for, as well as any areas containing repeated sequences, have very high depths in comparison with the areas of intergenic sequences (Figures 2 and 3). This poses a challenge for the assembly software, which is based on the assumption of uniform depth over the sequencing data (Miller, Koren, & Sutton, 2010).

Overall, the MN cluster seems to be more successfully assembled than the LA cluster, which is more fragmented (Figure 5). Differences in assembly completeness between the two regions might be a result of several factors. Firstly, the MN region has more flanking genes in closer proximity to the *Hb* region, which results in a higher density of probes. Secondly, the overall repeat content of the LA region is one order of magnitude larger than in the MN region, largely due to the larger proportion of simple repeats. Repeat content is a major interference in capture experiments because unwanted repetitive DNA may be enriched for, especially if there are repeated sequences included in the probe design. Furthermore, if probes were not completely covered by target DNA, they get single‐stranded sticky ends that can hybridize to repetitive or other nontarget DNA (Newman & Austin, 2016). Lastly, unless there were some longer reads that bridged such areas, this would in turn have led to gaps in the downstream de novo assemblies. Following that the assembly success was possibly a result of read length, we reason that a future increase of the average read length from 3 kbp to 5‐10 kbp would be sufficient to substantially increase the completeness of the assemblies. Due to the current circular consensus (CCS) PacBio sequencing technology, however, which is a trade‐off between accuracy and length of reads, longer reads with sufficient accuracy are not feasible.

### 3.4 Long‐read sequencing capture across species harbours new potential for comparative genomic studies

The number of reads mapping to the target regions was in the range of 23–43%, which may be seemingly low compared with other capture studies. For instance, a whole‐exome capture study on humans reported 56.1% of reads mapped to the target regions (Guo et al., 2012) and a similar study in rats reported to have 78.3% of reads on target (Yoshihara et al., 2016). In contrast to our study, however, these capture experiments enriched either the exome or ultra‐conserved elements within a single species, allowing for more efficient capture of conserved sequences. We were, however, able to cover up to 98% of the target regions with a sequencing depth of >10 reads across species (Table S1) which is similar to what mentioned experiments within human and rat exomes reported (Guo et al., 2012; Yoshihara et al., 2016) and the main difference is the higher percentage of nontarget sequences in our study.

We were able to capture complete genes for species with 70 My divergence time from the Atlantic cod (Figure 5). As expected, we found that capture success declines with increased sequence divergence between the reference genome of which we chiefly based our capture probes and the genomes of the included codfishes (Figure 4). It has been reported that orthologous exons were successfully captured in highly divergent frog species (with 200 My of separation); nevertheless, the capture success greatly decreased with increased evolutionary distance (Hedtke, Morgan, Cannatella, & Hillis, 2013). Similarly, it has been demonstrated that it is possible to capture >97% of orthologous sequences in four species of primates that diverged from humans 40 My ago, using probes entirely based on the human exome (George et al., 2011). Further, exomes were effectively captured from skink species that diverged up to 80 My from the reference, yet reporting a substantial decline in capture efficiency for sequences >10% different from the reference species (Bragg et al., 2015). Our study stands out from previous capture experiments because intergenic, noncoding sequences in addition to genes were captured across distantly related species. Efficient capture of intergenic sequences requires lower divergence time, as these regions usually evolve faster than genes (Koonin & Wolf, 2010). Thus, the most distantly related species from Atlantic cod for which we captured both coding and noncoding sequences was burbot, which diverged from Atlantic cod 46 My (Figure 5). We argue, in line with Schott et al. (2017), that sequence divergence may be a more exact predictor of capture success than evolutionary distance, as the sequence capture process is mainly influenced by the difference between the probe sequence and the target sequence. European hake, marbled moray cod and roughhead grenadier all diverged from cod about 70 My ago; however, the European hake *Hb* regions were more successfully captured and assembled (Table 1; Figure 2). This could be due to European hake having a lower genomewide divergence to Atlantic cod than marbled moray cod and roughhead grenadier (809k vs 879k and 907k SNPs; Table S1).

Finally, it should be mentioned that cusk—which diverged from Atlantic cod 39 My ago—was added to the experimental design after the probes were generated. Thus, the successful capture of cusk was therefore solely based on cross‐species target enrichment, demonstrating the power of heterologous probe targeting.

### 3.5 Concluding remarks and future perspectives

Here, we have successfully demonstrated that combining targeted sequence capture with long‐read sequencing technology is as an efficient approach to obtain high‐quality sequence data of a specific genomic region, including both coding and noncoding sequences, across evolutionary distant species. We show that genomewide divergence is of importance for capture success across species. Furthermore, the use of long‐read sequencing augmented the de novo assembly of regions containing repeated sequences that would otherwise fragment assemblies based on short‐read sequencing. This is crucial for capturing complete intergenic sequences that may be highly divergent compared to genic regions even among fairly closely related species. Given the rapid development in sequencing technologies, future methods will enable read‐through of repeated regions and thus further increase the completeness of assemblies. Moreover, a less stringent hybridization protocol should make it possible to capture sequences across even deeper evolutionary time. In sum, our approach has generated a cross‐species genomic resource across distantly related codfishes and shows the potential of enhancing comparative genomic studies of continuous genic and intergenic regions between any eukaryotic species‐group where genomic resources are scarce.

## 4 MATERIAL AND METHODS

### 4.1 Defining target regions and probe design

The probe design was chiefly based on the high‐quality genome of Atlantic cod, known as gadMor2 (Tørresen et al., 2017). In addition, species‐specific probes were designed based on low‐coverage assembled genomes (Malmstrøm et al., 2016, 2017) for ten selected species representing six families in the Gadiformes order. These species were Atlantic cod (*Gadus morhua*), Alaskan Pollock (*Gadus chalcogrammus*), polar cod (*Boreogadus saida*), haddock (*Melanogrammus aeglefinus*), Silvery pout (*Gadiculus argenteus*), burbot (*Lota lota*), European hake (*Merluccius merluccius*), roughhead grenadier (*Macrourus berglax*), roughsnout grenadier (*Trachyrincus scabrus*) and marbled moray cod (*Muraenolepus marmoratus*).

To retrieve relevant sequence data for the probe design, the MN and LA *Hb* regions were extracted from gadMor2 (Figure 1). These sequences, hereby known as the target regions, were then used as queries in BLAST (Altschul, Gish, Miller, Myers, & Lipman, 1990) searches with an E‐value threshold of <0.1 against the genome assembly data of all ten species.

In total, 5604 sequences from the chosen species were supplied to NimbleGen probe design. Protein‐coding genes from the ENSEMBL database were used to define the regions to be tiled in the probe design (Table S5) within the target regions of the Atlantic cod and the unitigs (i.e., high confidence contigs) for each of the additional codfishes.

NimbleGen SeqCap EZ capture probes were designed by NimbleGen (Roche, Madison, USA) using a proprietary design algorithm. NimbleGen offers an in‐solution sequence capture protocol, which includes custom‐made probes. Uniquely, the capture probes from NimbleGen are tiled to overlap the target area. 50–100 bp (average 75 bp) probes were designed tiled over the target regions (subset of gadMor2) resulting in each base, on average, being covered by two probes. Additionally, raw reads from Illumina sequencing from Malmstrøm et al. (2016, 2017) were used for each species to estimate repetitive sequences in each of the species genomes, aiming to discard probes containing any repeats. A more detailed description of the probe design is provided in Supporting Information Materials and Methods and Table S6.

### 4.2 Sample collection and DNA extraction

Our goal working with animals is always to limit any harmful effects of our research on populations and individuals. Whenever possible, we try to avoid animals being euthanized to serve our scientific purpose alone by collaborating with commercial fisheries or museums. The tissue samples used in this study are either from commercially fished individuals intended for human consumption or museum specimen. The commercially caught fish were immediately stunned, by bleeding following standard procedures by a local fisherman. There is no specific legislation applicable to this manner of sampling in Norway; however, it is in accordance with the guidelines set by the “Norwegian consensus platform for replacement, reduction and refinement of animal experiments” (www.norecopa.no).

DNA was extracted from tissue samples using high‐salt DNA extraction method by Phill Watts (https://www.liverpool.ac.uk/~kempsj/IsolationofDNA.pdf, last day accessed: December 2017). The concentration and purity of the DNA samples were quantified using NanoDrop (Thermo Fisher Scientific, Waltham, MA, USA) and a Qubit fluorometer (Invitrogen, Thermo Fisher Scientific, Waltham, MA, USA). Due to poor DNA quality, three species included in the probe design; Alaskan Pollock, polar cod and roughsnout grenadier were excluded from further analysis. In total, eight species were sequenced (Table S8); seven of these species were included in the probe design and one closely related species (cusk), which serves as a cross species capture experiment without species‐specific probes.

### 4.3 Capture, library preparation and sequencing

The sequencing libraries were prepared following a modified Pacific Biosciences SeqCap EZ protocol. As multiplexing of the samples before capture was required, barcodes were designed at the Norwegian Sequencing Centre (http://www.sequencing.uio.no) using guidelines from Pacific Biosciences (for more information see Supporting Information Materials and methods, Table S7 and Figure S3). Genomic DNA was sheared to 5‐kb fragments using MegaRuptor (Diagenode, Seraing (Ougrée), Belgium). Due to poorer DNA quality, fragmenting was not done for European hake. For this sample together with fragmented DNA from roughhead grenadier, short fragments were removed using BluePippin (Sage Science, Beverly, MA, USA) before library preparation. Illumina libraries were prepared using KAPA Hyper Prep Kit (Kapa Biosystems, Wilmington, MA, USA) and barcoded using different Illumina barcodes. PacBio barcodes were implemented during precapture amplification of libraries. After amplification, fragment length distribution was evaluated using Bioanalyzer (Agilent Technologies, Santa Clara, CA, USA) and samples were pooled in equimolar ratio. During hybridization, SeqCap EZ Developer Reagent (universal repeat blocker for use on vertebrate genomes) and oligos corresponding to Illumina and PacBio barcodes were used for blocking. Captured gDNA was amplified to ensure that sufficient amount of DNA was available for PacBio library preparation. Size selection of the libraries was performed using BluePippin. Final libraries were quality‐checked using Bioanalyzer and Qubit fluorometer (Invitrogen, Thermo Fisher Scientific, Waltham, MA, USA) and sequenced on RS II instrument (PacBio, Menlo Park, CA, USA) using P6‐C4 chemistry with 360 minutes movie time. In total, nine SMRT cells were used for sequencing.

### 4.4 De novo assemblies

Reads were filtered and demultiplexed using the “RS_reads of insert.1” pipeline on SMRT Portal (SMRT Analysis version smrtanalysis_2.3.0.140936.p2.144836). Each set of reads corresponding to a given species was cross‐checked with their respective six‐nucleotide Illumina adapter. Reads containing an incorrect Illumina adapter were removed. Adapter sequences were then trimmed using the application Prinseq‐lite v0.20.4 (Schmieder & Edwards, 2011). The trimmed reads were assembled de novo using Canu v1.4 + 155 changes (r8150 c0a988b6a106c27c6f993dfe586d2336282336a6) (Berlin et al., 2015). The Canu software is optimized for assembling single‐molecule high noise sequence data. We specified genome size as the size of the target regions (300 kbp). Additionally, we ran PBJelly (English et al., 2012) on the Canu de novo assemblies, using the raw reads to possible bridge gaps between scaffolds, settings given in Supporting Information Materials and Methods.

We assessed the assemblies by running Assemblathon 2 (Bradnam et al., 2013), which reports assembly metrics such as the longest contig, the number of contigs and the N50 value. De novo assemblies of the MN and LA regions of Atlantic cod and haddock were aligned and compared to their reference genomes, gadMor2 and melAeg, respectively, using BLAST and BWA v0.7.10 (Li & Durbin, 2009) to determine syntenic similarities and assembly completeness.

### 4.5 Estimating capture success

PacBio reads for all the species were mapped back to the Atlantic cod genome assembly (gadMor2) in order to determine sequence capture success and target mapping depths. Mapping was done using BWA‐MEM v0.7.10 (Li & Durbin, 2009). Target‐area read depth for all the species based on mapping against gadMor2 was calculated using Samtools v1.3.1 (Li et al., 2009). We calculated both average and median mapping depth against the target regions as a whole and for the MN and LA regions separately. We also calculated percentage of reads that mapped to the target regions and the percentage of the target regions covered by reads to a minimum depth of 10x. To compare assembled target regions, we additionally mapped the assemblies to the Atlantic cod target regions. In order to verify the sequence capture process, sequence data for Atlantic cod and haddock were mapped back to their reference genomes using BWA‐mem v0.7.10 (Li & Durbin, 2009). The results were visualized using Integrative Genome Viewer (Robinson et al., 2011).

To obtain an independent measure of divergence between species in the capture experiment, we calculated genomewide level of divergence of each species to the reference genome of Atlantic cod using low‐coverage whole‐genome sequence data from Malmstrøm et al. (2016, 2017). We mapped raw reads to Atlantic cod using BWA‐MEM (Li & Durbin, 2009) and called SNPs using the Freebayes variant caller (Garrison & Marth, 2012). Some species are more closely related to Atlantic cod than others, which could introduce a bias in mapping. To avoid this, we only looked at genomic regions where all species mapped. The number of SNPs was then used as an estimate of genomewide divergence of each species to Atlantic cod. We also mapped a low‐coverage genome of Atlantic cod to the Atlantic cod reference genome as a control.

In pursuance of factors explaining capture success, we tested for correlations and plotted the relationship between the genomewide level of divergence and the following variables; median mapping depth against the target regions (for total, LA and MN, respectively); percentage of reads that mapped to the target regions; and the percentage of the target regions covered by reads. All tests and plots were done using R version 3.2.5 (R Core Team, 2013).

Assembly continuity is very often hampered by the presence of repeats, which create gaps. We therefore quantified repeat content in the target regions extracted from gadMor2 and orthologous regions in haddock using Repeatmasker Open 3.0 (Smit, Hubley, & Green, 2010) for the MN region and the LA region separately.

### 4.6 Identifying gene location and synteny

In order to identify the genes of interest and their location in the assembly, we used local sequence alignment algorithm BLAST v2.4.0 (Altschul et al., 1990) with protein sequences of the genes of interest (Table S5) as queries. tblastn was used with an e‐value of 0.1. Investigation of *Hbb1‐Hba1* promoter region was done for four species, Atlantic cod, haddock, silvery cod and cusk. Sequences were aligned with ClustalW default settings using MEGA7 (Kumar, Stecher, & Tamura, 2016). Ancestral sequence reconstruction was carried out for *Hbb‐1* gene sequences from 24 species of codfishes from Baalsrud et al. (2017) using a maximum‐likelihood method implemented in MEGA7 (Kumar et al., 2016).

Additionally, we estimated sequence identity using EMBOSS Needle (Rice, Longden, & Bleasby, 2000) with default settings, between *Hbb* gene sequences from Baalsrud et al. (2017) that were missing and present in the de novo assemblies to evaluate similarity (Table S2).

## AUTHOR CONTRIBUTIONS

H.T.B. and S.J. initially conceived and designed the study, with input from S.N.K.H, A.T.‐K., M.S., G.O., R.S. and K.S.J. Tissue samples were provided by S.J. and H.T.B. Probe design was carried out by T.R. with assistance from S.N.K.H and H.T.B. DNA extraction and sequence library preparation were performed by S.N.K.H and A.T.‐K, respectively. Sequence capture was carried out by S.N.K.H, A.T.‐K., M.S. and G.O. Filtering, mapping of sequences and de novo assemblies were done by S.N.K.H., assisted by O.K.T and H.T.B. Annotation of genes, synteny analyses, statistical analyses and construction of all figures and tables were done by S.N.K.H and H.T.B. The manuscript was written by S.N.K.H and H.T.B. with input from S.J. and K.S.J.

## COMPETING INTERESTS

The authors declare that they have no competing interests.

## DATA AND MATERIALS AVAILABILITY

All reads and assemblies (unitigs) reported in this study plus the target regions (subset of gadMor2), the relevant sequence data for the probe design from the chosen species supplied to NimbleGen and the probe sequences have been deposited at figshare: https://doi.org/10.6084/m9.figshare.5875842.
