# Supplementary material for: Long‐read sequence capture of the haemoglobin gene clusters across codfish species
Source: Mol Ecol Resour. 2018 Dec 4;19(1):245–59. doi: 10.1111/1755-0998.12955 (PMC7379720; doi:10.1111/1755-0998.12955)
Supplement: Supplementary file 1 [file MEN-19-245-s001.pdf]

MOLECULAR ECOLOGY RESOURCES

Supplemental Information for:

Long-read sequence capture of the hemoglobin gene clusters across codfish species

Siv Nam Khang Hoff, Helle T. Baalsrud, Ave Tooming-Klunderud, Morten Skage, Todd Richmond, Gregor Obernosterer, Reza Shirzadi, Ole Kristian Tørresen, Kjetill S. Jakobsen and Sissel Jentoft[†]

Table of Contents:

**Supplementary Material and Methods**	Page 2-5
**Probe design**	Page 2-4
**PacBio barcoding and oligo design**	Page 4
**Read filtering**	Page 4
***De novo* assembly**	Page 4-5
**Mapping reads to the target regions**	Page 5
**Supplementary Tables S1 – S8**	Page 6-14
**Supplementary Figures S1 -S3**	Page 15-17
**References**	Page 18

Supplementary Material and Methods

Probe design

The initial target sequences from related species were identified using blastn (Altschul 1990) using the DNA sequence of the hemoglobin clusters in *Gadus morhua* as the query sequences as following:

```
ncbi-blast-2.2.31+/bin/tblastn -query
../Gadus_morhua_hemoglobin_proteins.fa -db MN_LA_candidates_dusted.fa
-out Hemoglobin_hits.txt -outfmt 6 -lcase_masking -num_threads 8
```

Candidate target regions for each species were then further refined by using tblastn and the protein sequences from the *Gadus morhua* hemoglobin genes and flanking genes as following:

```
ncbi-blast-2.2.31+/bin/tblastn -query
flanking_genes/Gadus_morhua.gadMor1.flanking_proteins.fa -db
MN_LA_candidates_dusted.fa -out Flanking_gene_hits.txt -outfmt 6 -
lcase_masking -num_threads 8
```

Hits from tblastn were then filtered to remove matches less than 20 amino acids in length, or with less than 65% identity. Each candidate region was then padded on each side by 10000 bases, resulting in candidate region totals as seen in Table S6.

Candidate probes of variable length, 50 to 100 nucleotides, were generated at a 5 bp step (5′ start to 5′ start). The length of each probe was determined by synthesis parameters, not taking into account GC content, Tm or other criteria. Rather than scoring target regions for repetitiveness, each individual candidate probe was scored for repeat content. Since complete genome sequence was not available for each of the target (draft) genomes, repeat scores were generated using WGS sequence from each species. WGS sequence read pairs (2x150 bp HiSeq) for each species were adapter trimmed and quality filter trimmed, removing leading or trailing bases where the base quality score was less than 20. Overlapping read pairs were merged using FLASH 1.2.8 software (ccb.jhu.edu/software/FLASH/), and then filtered by length, retaining only merged read pairs, and unmerged quality trimmed read pairs, which were at least 100 bp in length. Using those sequences, a 15-

mer histogram was created for each species using a random sampling of 4.2 Gbp of sequence, to represent 5X genome equivalents.
The repetitiveness of the probes was checked using each species histogram sequentially. The repeat score is defined as the average 15-mer frequency of the probe, determined by sliding a 15-mer window across the entire length of the probe and averaging the count of each 15-mer. For each probe, the highest repeat score across the 10 species was retained. Each probe was uniqueness checked against each of the 10 species individually, using the 5X genome equivalent reads as the "genome", using SSAHA version 1 (https://www.sanger.ac.uk/science/tools/ssaha). A genome match was defined as a stretch of sequence at least 30 bp in length, allowing up to 5 mismatches, insertions or deletions. The maximum number of matches for each probe across all each species was retained as the match value. For the 9 selected species, the match total was divided by 5 to compensate for the 5X genome equivalents that were screened. By storing the highest repeat score and the highest number of matches in the candidate probe database, the candidate probes for the capture design are biased to be very conservative.
For both *Gadus morhua* and the selected subset of codfishes, only unique probes (matches <= 1) were selected and used a very stringent threshold for the repeat score (average 15-mer frequency less than 25), to minimize the amount of off-target fragments that would be captured. Complete target coverage was deemed to be unnecessary, given the length of fragments to be captured, hence the attempt to use unique and non-repetitive probes. Capture probes were selected at an average spacing of 35 bp (5' start to 5' start). Candidate probes were ranked by score, based on uniqueness, repetitiveness, probe Tm, and base pair compositions, and the best scoring probe in each selection window (15 bp) was selected. The selection window was then advanced 20 bp and the process repeated until the end of the target sequence was reached. The average probe length is 75 bp, meaning that, on average, each base is covered by 2 capture probes. For *Gadus morhua*, 7057 probes were selected to cover the 337 kb of sequence. The selected probes cover 265 kb (78.7%) of the target directly, and 335 kb (99.4%) of the target would be estimated to be covered by captured fragments using an offset of 1000 bp. For the related species, 29774 probes were selected to cover the 1.82 Mbp of target sequence. The selected probes cover 1.27 Mbp (69.6%) of the targets directly and 1.818 (99.9%) of the target would be estimated to be covered by captured

fragments using an offset of 1000 bp. Each selected probe was represented on the final design a total of 57 times.

PacBio barcoding and oligo design

The PacBio barcoding and oligo design uses the the NimbleGen SeqCap EZ kit as template, which makes it possible to utilize the adapter kit for ligation- and blocking oligos during hybridization reactions. SeqCap EZ kit Oligo1 (yellow in figure S3) is similar to the first part of the Illumina Truseq universal adapter and Oligo2 is the reverse complement of the last bases in the Truseq indexed adapter, which make up the 3′of the Pre-capture oligos in addition to the PacBio barcode at its 5′ (green in figure S3). Pre-capture amplification add the PacBio barcode to the library, with unique barcodes at each end (asymmetric). The combined use of ligation indexing and PacBio barcodes is a robust cross check for sample identity (see main paper). The post capture oligos with the 3′end extending into the Illumina Truseq adapter, ensures that only fragments with a correct design are amplified (blue in Figure S3). The associated blocking oligos for the PacBio barcodes (red in Figure S3), as well as blocking reagents for the Illumina adapter region as part of the Seqcap EZ kit (HE-blocking in Figure S3), are needed for the hybridization reaction. List of oligos for pre- and post - capture amplification and associated blocking oligos for hybridization capture enclosed in Table S7.

Read filtering

Reads were filtered and de-multiplexed using the 'RS_reads of insert.1' pipeline on SMRT Portal (SMRT Analysis version smrtanalysis_2.3.0.140936.p2.144836) using following settings: Minimum number of passes = 0, minimum accuracy = 0.8, minimum barcode score = 24. Each set of reads corresponding to a given species was crossed-checked with their respective six-nucleotide Illumina adapter.

De novo assembly

We varied different options to optimize the assembly process to our data. The options yielding best results and the option that was chosen for the final assembly was the following corOutCoverage=500, corMhapSensitivity=high, corMinCoverage=0, minOverlapLength=200, and corMaxEvidenceErate=0.15.

We assembled the target regions *de novo* for each of the eight codfishes using Canu v1.4 +155 changes (r8150 c0a988b6a106c27c6f993dfe586d2336282336a6)(Berlin *et al.* 2015) varying the

following options, corOutCoverage by 500, 600, 1000, 2000, and minOverlapLength by 100, 200, and to 500. The setting corOutCoverage set to 500 and minOverlapLength set to 200 gave us the longest unitigs, while retaining maximum number of *Hb* and flanking genes in our assemblies. Following options where used:
canu -p <filename> -d <dirname> genomeSize=0.3m corMhapSensitivity=high corOutCoverage=500 corMinCoverage=0 minOverlapLength=200 corMaxEvidenceErate=0.15 maxThreads=5 contigFilter="2 1000 1.0 1.0 2" -pacbio-raw <raw_reads.fastq>

Furthermore, Pbjelly (English *et al.* 2012) was used with raw reads as input to fill possible gaps using the following options:
<jellyProtocol>
<reference>/assembly.fasta</reference>
<outputDir>/species</outputDir>
<blasr>-minMatch 8 -minPctIdentity 70 -bestn 1 -nCandidates 20
-maxScore -500 -nproc 10 -noSplitSubreads</blasr>
<input baseDir="/raw_reads_directory/">
<job>raw_reads.fastq</job>
</input>
</jellyProtocol>

Mapping reads to the target regions

PacBio reads for all species were mapped back to the Atlantic cod reference genome, gadMor2 (Tørresen *et al.* 2017) in order to determine sequence capture success and target mapping depths. Mapping was done using BWA-mem v0.7.10 (Li & Durbin 2009) with the following options: -k17 -W40 -r10 -A2 -B5 -O2 -E1 -L0.

Supplementary Tables

Table S1: For each species, the average and median depth of reads mapped against the target regions (for MN, LA and total), the genomic divergence to Atlantic cod (number of SNPs), percentage of nucleotides mapped to the target and the percentage of the target regions with more than 10x coverage.

Species	Average mapping depth			Median mapping depth			Genomic divergence to Atlantic cod	Percentage of nucleotides mapped	Percentage of target regions covered by >10x
	MN	LA	Tot	MN	LA	Tot	SNPs		
**Atlantic cod**	277	230	260	266	203	242	75369	43	100
**Haddock**	122	100	114	106	79	97	322534	40	98
**Silvery pout**	130	161	142	73	93	80	562937	27	93
**Cusk**	160	128	149	126	92	114	587271	34	95
**Burbot**	159	136	150	148	97	124	639069	36	91
**European hake**	129	102	119	80	37	56	809403	27	77
**Marbled moray cod**	77	87	81	21	18	20	879212	23	63
**Roughhead grenadier**	80	70	77	14	8	12	906578	25	53

Table S2: Estimated sequence identity using EMBOSS Needle (Rice *et al.* 2000) with default settings, between paralogous *Hbb* gene sequences from Baalsrud *et al.* 2017. Genes highlighted in bold are missing from the assemblies in figure 5.

Species	Genes compared	Sequence identity
**Silvery pout**	*Hbb2* – ***Hbb3***	433/441 (98.2%)
**Roughhead grenadier**	*Hbb2* – ***Hbb3***	373/442 (84.4%)
**Cusk**	*Hbb1* – ***Hbb2***	316/470 (67.2%)
**Burbot**	*Hbb1* – ***Hbb2***	320/485 (66.0%)

Table S3: Amount of repeated sequences in the target regions of the Atlantic cod (Tørresen *et al.* 2017; gadMor2) and haddock (Tørresen *et al.* 2018; melAeg) given in percentage.

	**MN**		**LA**	
	Atlantic cod	Haddock	Atlantic cod	Haddock
**Retro elements**	1.0	4.11	2.8	3.98
**Transposons**	1.3	3.17	2.4	3.44
**Simple repeats**	5.8	7.8	13.8	12.02
**Low complexity and unclassified repeats**	2.6	1.48	1.3	1.38
**Total repeated sequences**	10.7	16.31	20.3	19.98

Table S4: Amino acids at positions 55 and 62 in Hbb1 in various codfishes taken from Baalsrud *et al.* 2017.

**Species**	**Pos 55**	**Pos 62**
Gadus morhua	V	A
Arctogadus glacilis	M	Q
Boreogadus saida	V	N
Trisopterus minutus	M	K
Pollachius virens	V	K
Melanogrammus aeglefinus	V	K
Merlangius merlangus	V	R
Theragra chalcogramma	M	K
Gadiculus argentus	M	Q
Phycis phycis	A	Q
Molva molva	M	K
Lota lota	L	K
Brosme brosme	M	K
Merluccius merluccius	M	K
Merluccius capensis	M	K
Merluccius polli	M	K
Melanonus zugmayeri	M	K
Macrourus berglax	L	K
Malacocephalus occidentalis	K	Q
Bathygadus melanobranchus	Q	K
Muraenolepis marmoratus	Q	N
Bregmaceros cantori	M	Q
Laemonema laureysi	M	R
Thrachyrincus scabrus	K	K

Table S5: Genes provided Nimblegen for the probe design and used to identify genes in *de novo* assemblies. For each gene, the gene name is given with its ENSEMBL name and ENSEMBL identifier.

Gene name	ENSEMBL name	ENSEMBL Gene ID
c17orf28	UBALD1B	ENSGMOG00000015870
foxj1a	FOXJ1	ENSGMOG00000015882
rhbdfb	RHBDF1	ENSGMOG00000017919
aqp8	AQP8A	ENSGMOG00000015825
aqp8	AQP8A	ENSGMOG00000001220
lcmt	LCMT1	ENSGMOG00000001230
arhgap17	ARHGAP8	ENSGMOG00000001252
polr3k	polr3k	ENSGMOG00000004334
mgrn1	mgrn1b	ENSGMOG00000004288
aanat	aanat2	ENSGMOG00000004277
rhbdf1a	rhbdf1b	ENSGMOG00000004239
mpg	mpg	ENSGMOG00000004225
nprl3	nprl3	ENSGMOG00000004199
kank2	kank2	ENSGMOG00000003897
dock6	dock6	ENSGMOG00000003777
elavl3	elavl3	ENSGMOG00000003760
prkcsh	prkcsh	ENSGMOG00000003727
hbz	HBZ	ENSGMOG00000017953
Hemoglobin beta 3		ENSGMOG00000020266
Hemoglobin alpha		ENSGMOG00000005709
Hemoglobin alpha1		ENSGMOP00000004430
Hemoglobin beta 2		ENSGMOP00000021753
Hemoglobin zeta		ENSGMOG00000017953.1
Hemoglobin alpha 3		ENSGMOG00000003938.1
Hemoglobin		ENSGMOG00000020497
Hemoglobin beta 2		ENSGMOG00000020266
Hemoglobin beta 3		ENSGMOG00000015840.1
Hemoglobin beta		ENSGMOG00000015832
Hemoglobin beta5		ENSGMOG00000019722

Table S6: Each candidate region for probe design and number of bases for each of the selected codfish species

Species	Number of Regions	Number of Bases
Boreogadus saida	47	177632
Gadiculus argentus	57	208963
Lota lota	42	284866
Macrourus berglax	26	137930
Melanogrammus aeglefinus	44	184666
Merluccius merluccius	37	193995
Muraenolepis marmoratus	50	196574
Theragra chalcogramma	45	201877
Trachyrincus scabrus	38	234180
**Grand Total**	**386**	**1820683**

Table S7: List of oligos for pre- and post – capture amplification and associated blocking oligos for hybridization capture used in this study.

Name	Sequence (5´-3´)
PreCap-2 fwd	GTCAGACGATGCGTCATAATGATACGGCGACCACCGAGA
PreCap-4 fwd	GCTATACATGACTCTGCAATGATACGGCGACCACCGAGA
PreCap-5 fwd	GTACTAGAGTAGCACTCAATGATACGGCGACCACCGAGA
PreCap-6 fwd	GTGTGTATCAGTACATGAATGATACGGCGACCACCGAGA
PreCap-7 fwd	GACACGCATGACACACTAATGATACGGCGACCACCGAGA
PreCap-12 fwd	GATGATGTGCTACATCTAATGATACGGCGACCACCGAGA
PreCap-13 fwd	GCTGCGTGCTCTACGACAATGATACGGCGACCACCGAGA
PreCap-14 fwd	GCGTCTATATACGTATAAATGATACGGCGACCACCGAGA
PreCap-16 fwd	GCATCACTACGCTAGAAATGATACGGCGACCACCGAGA
PreCap-2 rev	CGCGATCTATGCACACGCAAGCAGAAGACGGCATACGAG
PreCap-4 rev	CGACTCTGCGTCGAGTCCAAGCAGAAGACGGCATACGAG
PreCap-5 rev	CTACAGCGACGTCATCGCAAGCAGAAGACGGCATACGAG
PreCap-6 rev	CGCGCAGACTACGTGTGCAAGCAGAAGACGGCATACGAG
PreCap-7 rev	CGTCTCTGCGATACAGCCAAGCAGAAGACGGCATACGAG
PreCap-12 rev	CACACTGACGTCGCGACCAAGCAGAAGACGGCATACGAG
PreCap-13 rev	CATAGAGACTCAGAGCTCAAGCAGAAGACGGCATACGAG
PreCap-14 rev	CCATAGCGACTATCGTGCAAGCAGAAGACGGCATACGAG
PreCap-16 rev	CTATGTGATCGTCTCTCCAAGCAGAAGACGGCATACGAG
Block-2 fwd	GTCAGACGATGCGTCAT
Block-4 fwd	GCTATACATGACTCTGC
Block-5 fwd	GTACTAGAGTAGCACTC
Block-6 fwd	GTGTGTATCAGTACATG
Block-7 fwd	GACACGCATGACACACT
Block-12 fwd	GATGATGTGCTACATCT
Block-13 fwd	GCTGCGTGCTCTACGAC
Block-14 fwd	GCGTCTATATACGTATA
Block-16 fwd	GCATCACTACGCTAGA
Block-2 rev	CGCGATCTATGCACACG
Block-4 rev	CGACTCTGCGTCGAGTC
Block-5 rev	CTACAGCGACGTCATCG
Block-6 rev	CGCGCAGACTACGTGTG
Block-7 rev	CGTCTCTGCGATACAGC
Block-12 rev	CACACTGACGTCGCGAC
Block-13 rev	CATAGAGACTCAGAGCT
Block-14 rev	CCATAGCGACTATCGTG
Block-16 rev	CTATGTGATCGTCTCTC
PostCap-2 fwd	GTCAGACGATGCGTCATAATGA
PostCap-4 fwd	GCTATACATGACTCTGCAATGA
PostCap-5 fwd	GTACTAGAGTAGCACTCAATGA
PostCap-6 fwd	GTGTGTATCAGTACATGAATGA
PostCap-7 fwd	GACACGCATGACACACTAATGA

PostCap-12 fwd	GATGATGTGCTACATCTAATGA
PostCap-13 fwd	GCTGCGTGCTCTACGACAATGA
PostCap-14 fwd	GCGTCTATATACGTATAAATGA
PostCap-16 fwd	GCATCACTACGCTAGAAATGA
PostCap-2 rev	CGCGATCTATGCACACGCAAGC
PostCap-4 rev	CGACTCTGCGTCGAGTCCAAGC
PostCap-5 rev	CTACAGCGACGTCATCGCAAGC
PostCap-6 rev	CGCGCAGACTACGTGTGCAAGC
PostCap-7 rev	CGTCTCTGCGATACAGCCAAGC
PostCap-12 rev	CACACTGACGTCGCGACCAAGC
PostCap-13 rev	CATAGAGACTCAGAGCTCAAGC
PostCap-14 rev	CCATAGCGACTATCGTGCAAGC
PostCap-16 rev	CTATGTGATCGTCTCTCCAAGC

Table S8:
Information on all the fish samples used in this study. We used one individual of each species (N = 1). Distribution and Depths information taken from fishbase.org (Eschemeyer & Fricke 2017).

Species	Year	Location	Tissue	Depth	Distribution
**Atlantic cod** ***Gadus morhua***	2009	North East Atlantic Ocean	spleen	Range 0 – 600m but usually found at 150 – 200m	North Atlantic and Arctic oceans
**Haddock** ***Melanogrammus aeglefinus***	2009	Lofoten, Norway	muscle/ spleen	10 – 450m, usually found 10 – 200m	Northeast Atlantic: Bay of Biscay to Spitzbergen; in the Barents Sea to Novaya Zemlya; around Iceland; rare at the southern Greenland. Northwest Atlantic: Cape May, New Jersey to the Strait of Belle Isle.
**Silvery pout** ***Gadiculus argentus***	2009	Inner Oslo Fjord, Norway	spleen	100 – 1000m	Northeast Atlantic: found in the western Mediterranean and in the Atlantic around the Strait of Gibraltar and to the south along the Moroccan coast.
**Cusk** ***Brosme brosme***	2011	Lofoten, Norway	spleen	150 – 450m	Northwest Atlantic: New Jersey to the Strait of Belle Isle and on the Grand Banks of Newfoundland. Rare at the southern tip of Greenland. Northeast Atlantic: off Iceland, in the northern North Sea, and along the coast of Scandinavia to the Murmansk Coast and at Spitzbergen.
**Burbot** ***Lota lota***	2012	Schwentine, Germany	spleen	Lives at the bottoms of rivers and lakes at 0.5 – 230m	Holarctic in freshwater.
**European hake** ***Merluccius merluccius***	2009	Inner Oslo Fjord, Norway	spleen	70 – 370m	Atlantic coast of Europe and western North Africa; northward to Norway and Iceland, southward to Mauritania. Also found in the Mediterranean Sea and along the southern coast of the Black Sea.
**Marbled moray cod** ***Muraenolepis marmoratus***	2006/ 2007	Elephant Isl. and the South Shetland Isl.	fin clip	30 – 1600m	Southern Ocean: known only from the Crozet, Kerguelen and Heards islands.

**Roughhead grenadier *Macrourus berglax***	2011	North East Atlantic Ocean	fin clip	100 – 1000m, usually found at 300 – 500m	North Atlantic Ocean

Supplementary Figures

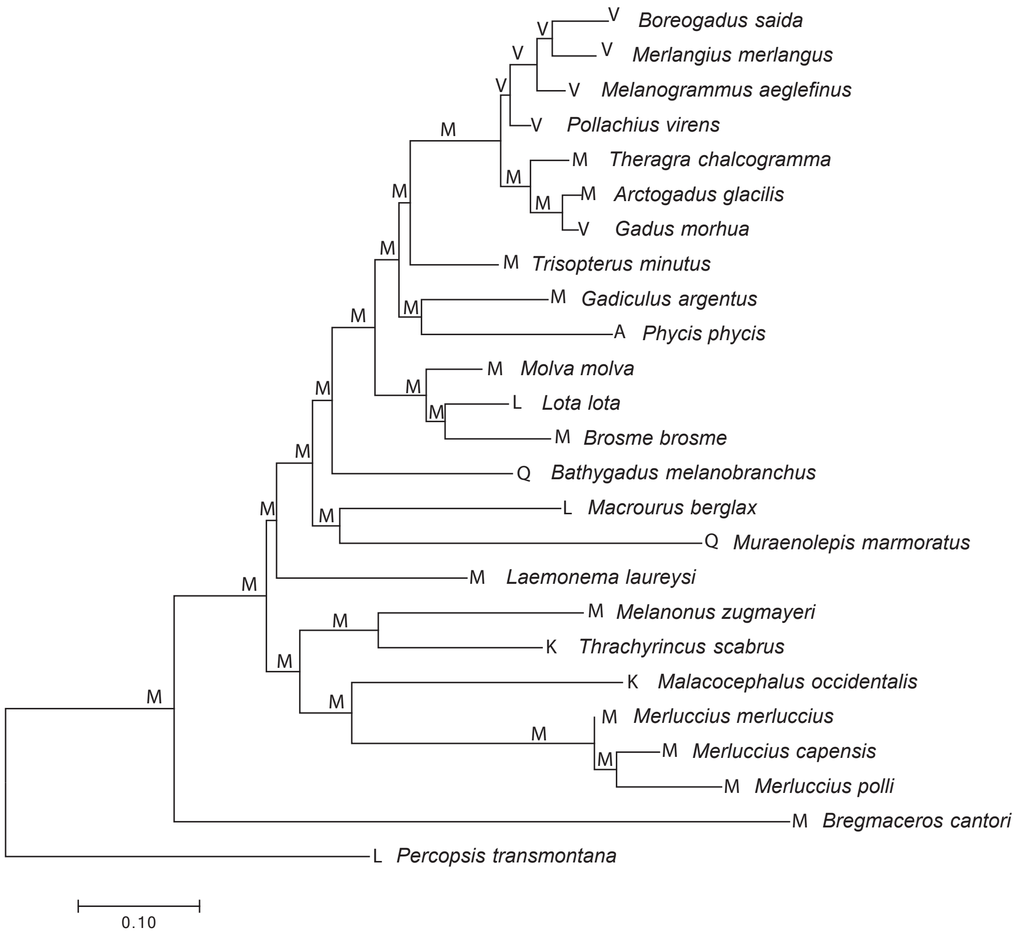

Figure S1: Ancestral reconstruction of amino acids at position 55 in the *Hbb-1* gene in Gadiformes. Phylogenetic trees and ancestral reconstruction were carried out in MEGA 7.0 (Kumar *et al.* 2016).

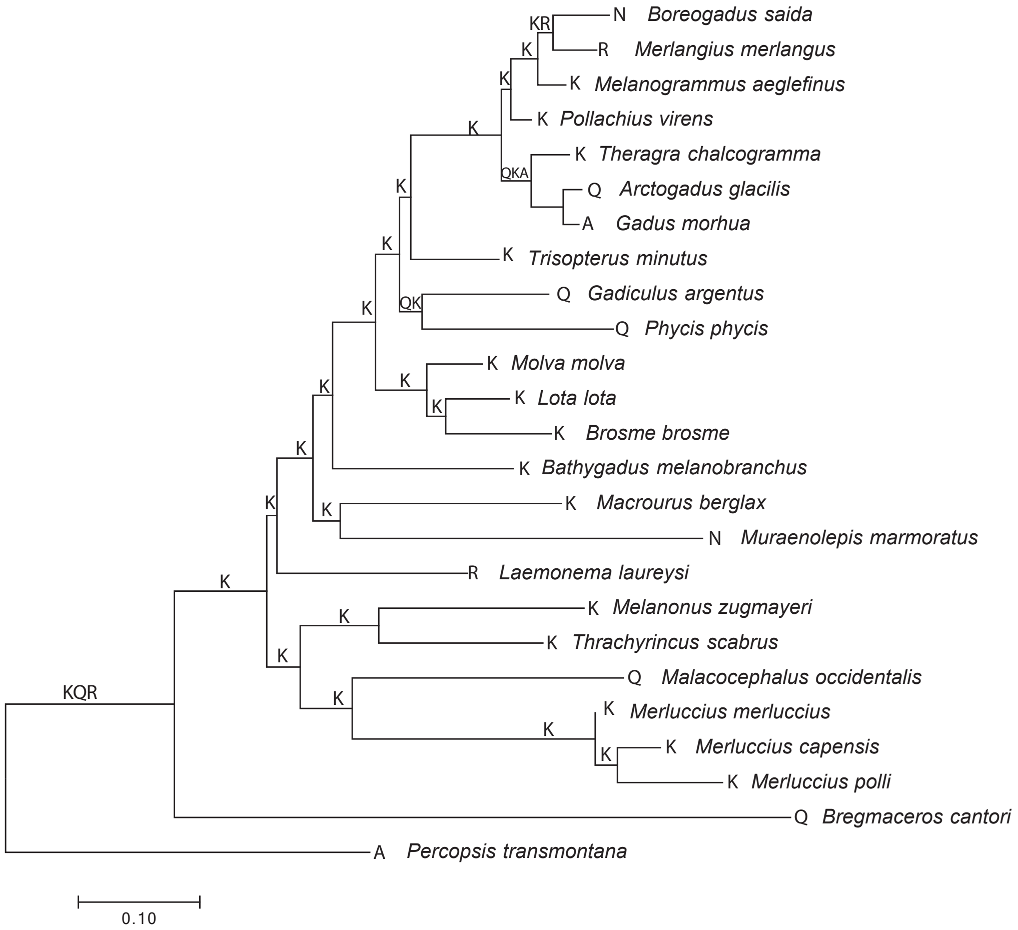

Figure S2: Ancestral reconstruction of amino acids at position 62 in the *Hbb-1* gene in Gadiformes. Phylogenetic trees and ancestral reconstruction were carried out in MEGA 7.0 (Kumar *et al.* 2016).

Name	5´ Sequence 3´
SeqCap Oligo1:	AATGATACGGCGACCACCGAGA
PreCap-2 fwd:	GTCAGACGATGCGTCATAATGATACGGCGACCACCGAGA……
Block-2 fwd:	GTCAGACGATGCGTCAT ------HE blocking from kit----------
PostCap-2 fwd:	GTCAGACGATGCGTCATAATGA
PreCap-2 rev:	CGCGATCTATGCACACGCAAGCAGAAGACGGCATACGAG…..
SeqCap Oligo2 :	CAAGCAGAAGACGGCATACGAG
Block-2 rev:	CGCGATCTATGCACACG ------HE blocking from kit----------
PostCap-2 rev:	CGCGATCTATGCACACGCAAGC

Figure S3: Example of the barcode and oligo design for the PacBio target enrichment assay used in this study. It involves adding unique PacBio barcodes (green) at each end of an Illumina compatible ligated and indexed library (yellow) by PCR.
Barcoded libraries are then pooled in equimolar concentrations before capture, with the use of blocking oligos (red, HE-blocking), followed by a final post capture amplification to boost library yield (blue).

References

Altschul, S. F., Gish, W., Miller, W., Myers, E. W., & Lipman, D. J. (1990). Basic local alignment search tool. *Journal of Molecular Biology*, *215*(3), 403–410. http://doi.org/https://doi.org/10.1016/S0022-2836(05)80360-2

Baalsrud, H. T., Voje, K. L., Tørresen, O. K., Solbakken, M., Matschiner, M., Malmstrøm, M., ... Jentoft, S. (2017). Evolution of hemoglobin genes in codfishes influenced by ocean depth. *Scientific Reports*, 7, 1–10. http://doi.org/10.1038/s41598-017-08286-2

Berlin, K., Koren, S., Chin, C.-S., Drake, J. P., Landolin, J. M., & Phillippy, A. M. (2015). Assembling large genomes with single-molecule sequencing and locality-sensitive hashing. *Nature Biotechnology*, *33*(6), 623–630. http://doi.org/10.1038/nbt.3238

English, A. C., Richards, S., Han, Y., Wang, M., Vee, V., Qu, J., … Gibbs, R. A. (2012). Mind the gap: upgrading genomes with Pacific Biosciences RS long-read sequencing technology. *PLoS ONE*, *7*(11), e47768–12. http://doi.org/10.1371/journal.pone.0047768

Eschemeyer, W. N., & Fricke, R. (2017). Catalog of fishes. http://researcharchive.calacademy.org/research/ichthyology/catalog/fishcatmain.asp. (Last accessed September 2018).

Kumar, S., Stecher, G., & Tamura, K. (2016). MEGA7: molecular evolutionary genetics analysis version 7.0 for bigger datasets. *Molecular Biology and Evolution*, *33*(7), 1870–1874. http://doi.org/10.1093/molbev/msw054

Li, H., & Durbin, R. (2009). Fast and accurate short read alignment with Burrows-Wheeler transform. *Bioinformatics*, *25*(14), 1754–1760. http://doi.org/10.1093/bioinformatics/btp324

Rice, P., Longden, I., & Bleasby, A. (2000). EMBOSS: The European molecular biology open software suite. *Trends in Genetics*, *16*(6), 276–277. http://doi.org/10.1016/S0168-9525(00)02024-2

Tørresen, O. K., Brieuc, M. S. O., Solbakken, M. H., Sørhus, E., Nederbragt, A. J., Jakobsen, K. S., ... Jentoft, S. (2018). Genomic architecture of haddock (*Melanogrammus aeglefinus*) shows expansions of innate immune genes and short tandem repeats. *BMC Genomics*, *19*(1), 51. http://doi.org/10.1186/s12864-018-4616-y

Tørresen, O. K., Star, B., Jentoft, S., Reinar, W. B., Grove, H., Miller, J. R., ... Nederbragt, A. J. (2017). An improved genome assembly uncovers prolific tandem repeats in Atlantic cod. *BMC Genomics*, *18*(1), 95. http://doi.org/10.1186/s12864-016-3448-x
